# Comparison of the Sorption Kinetics of Lead(II) and Zinc(II) on Titanium Phosphate Ion-Exchanger

**DOI:** 10.3390/ijms21020447

**Published:** 2020-01-10

**Authors:** Marina V. Maslova, Vladimir I. Ivanenko, Nataliya Yu. Yanicheva, Natalia V. Mudruk

**Affiliations:** Tananaev Institute of Chemistry—Subdivision of the Federal Research Centre “Kola Science Centre of the Russian Academy of Sciences” (ICT KSC RAS), «Academic town» 26a, Apatity 184209, Murmansk region, Russia; v.ivanenko@ksc.ru (V.I.I.); mage13@bk.ru (N.Y.Y.); kirichnv@gmail.com (N.V.M.)

**Keywords:** titanium phosphate, ion-exchanger, heavy metals, kinetics, radius of adsorbed ion, effective diffusion coefficient

## Abstract

The treatment of heavy metal-contaminated wastewater is an important action to reduce the negative impacts of industrial wastes on water bodies. This work focuses on the application of a low-cost titanium (IV) phosphate sorbent of TiO(OH)H_2_PO_4_·2H_2_O chemical composition toward lead and zinc ions depending on their concentration and the temperature of the solution. The kinetic studies showed that the values of the rate of intraparticle diffusion and the effective diffusion coefficients for Zn^2+^ were considerably higher than those for Pb^2+^. To explain the difference between the sorption kinetics rates for Pb^2+^ and Zn^2+^, the effective radius and dehydration degree of the adsorbed ions were calculated. The sorbent capability of the lead and zinc ion removal and its excellent efficiency in the presence of a high concentration of calcium ions were demonstrated using simulated mine water. Due to the fast kinetics and the high exchange capacity of titanium phosphate toward divalent ions, this sorbent can be considered as a promising material for the concentration and immobilization of heavy metals into the phosphate matrix.

## 1. Introduction

Heavy metals are known to be among the priority environmental pollutants [1,2]. The treatment of wastewater polluted by heavy metals is an important action to reduce the negative impacts of industrial wastes on water bodies. Wastewater treatment is also essential for the safety and quality management of drinking water. It should be noted that lead is one of the most toxic elements, and it is poisonous to living organisms at certain concentrations [3]. It usually occurs along with zinc in lead–zinc mine water and the wastewater of many industries, such as electroplating, textile mills, and manufacturing of metals, paints, viscose fibers, and chemicals [4].

Currently, the most common method of removing lead and zinc ions from wastewater is chemical precipitation [5]. This process is simple, but it depends on factors such as the temperature, the presence of impurities in the solution (which impede the ion precipitation), and concentrations of metal ions. These toxic metals have very low threshold limit values (TLV) (0.05 mg·L^−1^ for Pb^2+^ and 2 mg·L^−1^ for Zn^2+^). This means that the process of chemical precipitation does not allow achieving acceptable levels of pollutants for safe discharge of wastewaters into nature. Moreover, chemical precipitation results in the formation of large volumes of sludge, as secondary waste, which, in turn, requires recycling. Using membrane processes for heavy metal removal gives better and more reliable results regarding water purification efficiency. However, the short membrane lifetime and fouling are severe drawbacks for this method [6,7]. The main disadvantage of electrochemical methods is the high power consumption [8].

Recently, sorption methods (adsorption and ion exchange) were widely used for natural water and wastewater treatment [9]. These methods are well-controlled processes, and they effectively remove many different types of impurities, regardless of their chemical resistance.

Studies carried out in the last few years showed that more than 70 natural and synthetic sorbents can be used to remove contaminants from aquatic environments [10]. However, when using mineral sorbents, it is not always possible to achieve reproducible results due to the unstable chemical composition and particle sizes of such materials. On the contrary, synthetic sorbents give more reliable results in wastewater treatment. They also have longer lifetimes due to their capability to reuse.

Among inorganic ion-exchangers, titanium phosphates (TiPs) represent a promising candidate for application in the field of wastewater treatment [11,12]. They have a wide range of valuable purification applications, especially in the processing of radioactive effluents and wastewater treatment [13,14,15]. Acidic titanium phosphates containing dihydrogen phosphate groups (TiHP) are of principal interest. Due to the presence of –H_2_PO_4_^2^^−^ groups forming strong acid sites, the titanium phosphates can be used at low pH values. Although ion-exchangers based on titanium phosphates are numerous, there are several compounds containing only dihydrogen phosphate groups: Ti_2_O_3_(H_2_PO_4_)·2H_2_O [16] and TiO(OH)(H_2_PO_4_)·2H_2_O [17,18]. Typically, TiHPs are synthesized either by precipitating from titanium(IV) salt solutions or through the treatment of titanium dioxide with the orthophosphoric acid. The synthesis of these materials is rather complicated; it is a long, multistep process requiring rigid synthesis conditions, high reagent consumption, elevated temperatures, autoclave equipment, and organic templates.

The wastewater treatment technique should not be costly; thus, the prices of sorbents play a major role. From this point of view, a new simpler method of TiHP production is definitely welcome. This work focuses on the applications of a low-cost titanium(IV) phosphate sorbent, which was synthesized via a novel minimalistic approach using a new crystalline precursor, (NH_4_)_2_TiO(SO_4_)_2_·H_2_O (ATS) [19]. This salt can be produced as a by-product from different titanium-containing ores. In our study, ATS was obtained according to the procedure by Gerasimova et al. [20] from mineral titanite (CaTiSiO_5_), which is an unprocessed industrial waste of apatite–nepheline ore processing. In contrast to the current solution-based preparations, the interaction between the crystalline titanium salt and phosphoric acid provides easy fabrication of the ion-exchanger, TiO(OH)H_2_PO_4_·2H_2_O, with acid –H_2_PO_4_^2−^ functional groups. Application of crystalline ATS as a titanium source made it possible to significantly diminish the number of synthesis stages and effluents, use a stoichiometric amount of dilute phosphoric acid, and obtain titanium phosphate of required structural type via an environmentally friendly technique.

When using the sorption process in practice, the kinetic features of the sorbent should be taken into account to determine the degree of purification as a function of the contact time between liquid and solid phases. Knowledge of the rate-controlling stage is a critical factor for selecting optimum operating conditions for the full-scale process, and it gives important information for designing the sorption process.

Commonly, the sorption kinetics depends on the initial concentration of solutes, particle size, and textural properties of a sorbent which determine the sizes of the solvated ions that may enter the sorbent matrix [21]. A literature review shows that the mass transfer stages, especially external diffusion, are not considered thoroughly when the kinetic properties of titanium phosphates are studied. The authors’ attention is usually focused on the study of the fourth stage (sorption) using pseudo-first- or pseudo-second-order reactions models [13,22,23]. At the same time, diffusion can also have a significant effect on the overall rate of the sorption process. Knowing the stage which inhibits mass transfer is necessary for selecting the optimal conditions of the sorption process, for example, hydrodynamic parameters, sorbent granule size, temperature, etc.

The objective of the work was to study the ability of the new TiP for the removal of lead and zinc ions from aqueous solutions. To evaluate the sorption properties of the new titanium phosphate, it was important to compare the sorption kinetics of ions with different crystal radius for understanding the role of ion-hydration effects in the sorption specificity. The influence of the adsorbed ion hydration on sorption is given extremely insufficient attention.

Therefore, this work focuses on the study of sorption kinetics of lead and zinc ions on titanium phosphate, including external and internal mass transfer, as well as chemical adsorption. The effect of the solute concentration and the temperature of the solution on the sorption kinetics was shown. For the first time, ion-exchange reaction rates at the external diffusion stage, activation energy, and effective diffusion coefficients were calculated. To understand the mechanism of the sorption kinetics, the radius and dehydration degree of the adsorbed ions, and also the activation energy were calculated. Thus, it is shown that the calculation of the dehydration degree of adsorbed ions allows predicting their kinetic behavior and sorption ability. The obtained results were confirmed by testing the sorbent on simulated mine water, contaminated with toxic metal ions.

## 2. Results

### 2.1. Characterization of the Sorbent

The elemental analysis data of the obtained solids are presented in Table 1. The experimental data collected for as-synthesized titanium phosphate (TiHP) yielded a composition of 38.2% ± 0.05% TiO_2_ and 33.8% ± 0.05% P_2_O_5_, which corresponds to a molar ratio of TiO_2_:P_2_O_5_ = 1:0.5. The theoretical TiO_2_ and P_2_O_5_ values calculated for the TiO(OH)(H_2_PO_4_)·2H_2_O formula were 37.4% and 33.2%, respectively, which gave acceptable differences of less than 2% when compared to the experimental data.

**Figure 1:**
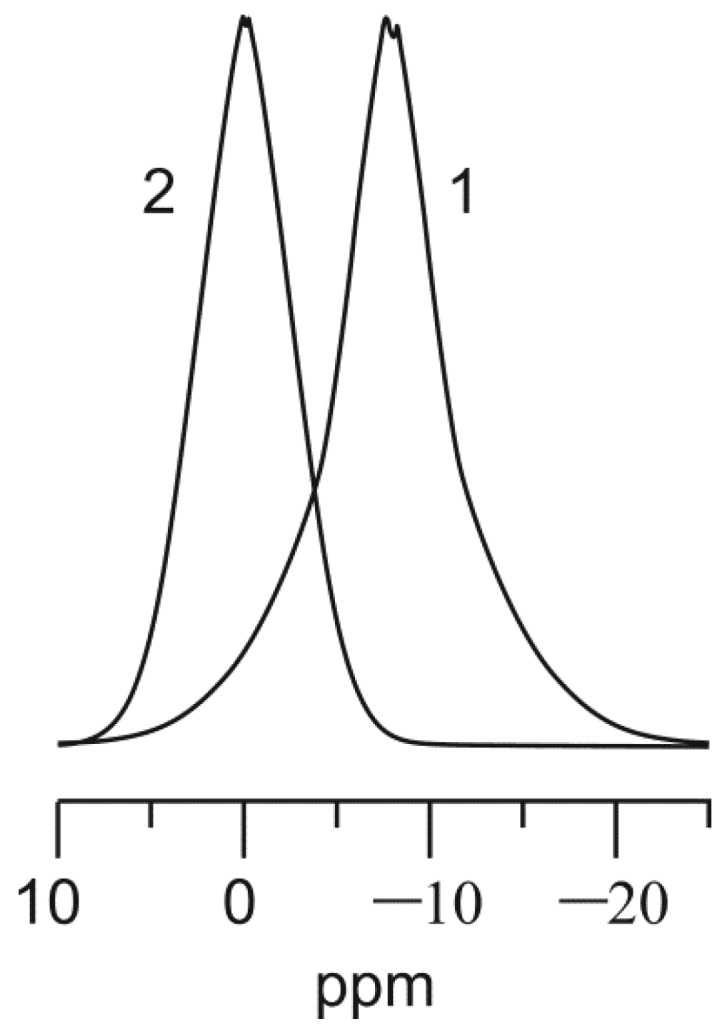
Single-pulse ^31^P-MAS NMR spectra of powder samples of TiHP (**1**), and Na-TiHP (**2**).

Figure 1 shows the spectrum of the magic-angle spinning nuclear magnetic resonance (^31^P-MAS NMR) data obtained for different TiP products all dried at 60 °C before the NMR experiments. A relatively broad peak (−25 ± 10 ppm) was observed due to the amorphous nature of phosphate framework. The main resonance lines at −7.4 ppm and −7.8 ppm for TiHP could be assigned to the presence of H_2_PO_4_^−^ species [17].

**Figure 2:**
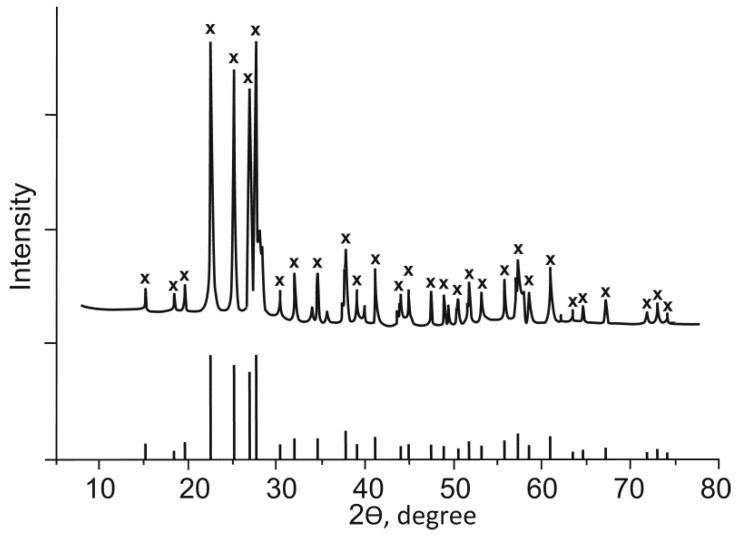
X-ray diffraction (XRD) powder patterns of the product calcined at 750 °C (**x** refers to the Ti_2_O(PO_4_)_2_ according to card PDF number 36-0699 [24]).

The calcination of TiHP at 750 °C resulted in the formation of a phase with the composition Ti_2_O(PO_4_)_2_ (Figure 2).

**Figure 3:**
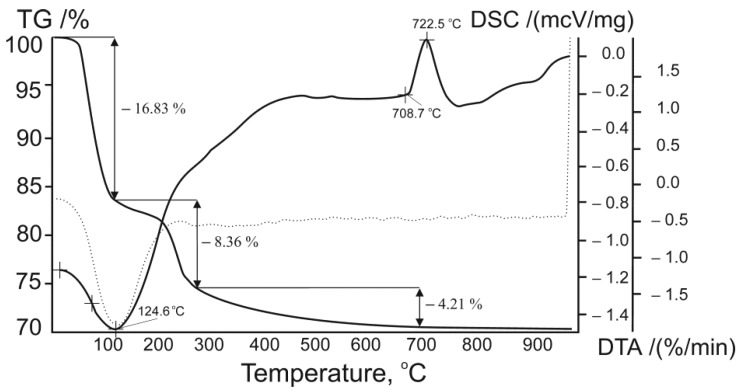
Thermogravimetric and differential scanning calorimetric (TG-DSC) data for obtained titanium phosphate.

According to the thermogravimetric curve of titanium phosphate (Figure 3), weight losses of 16.67% and 4.28% were observed in the regions of 25–125 °C and 125–280 °C, respectively. A broad endothermic peak occurred at 124.6 °C due to the removal of adsorbed and coordinated water molecules. In the temperature range of 125–280 °C, two concurrent processes took place: the condensation of hydroxo- and hydrogen phosphate groups [25]. Above 280 °C, the mass loss was 4.21%. No mass loss was observed at temperatures above 700 °C, and an exothermic peak was observed at 722 °C corresponding to the titanium phosphate transformation into the Ti_2_O(PO_4_)_2_ phase according to the thermolysis process [18].

$$\begin{array}{l} 2TiO\left(OH\right)H_{2}PO_{4}\cdot 2H_{2}O\frac{-4H_{2}O}{20-135^{\circ }C}2TiO\left(OH\right)H_{2}PO_{4}\frac{-2H_{2}O}{130-285^{\circ }C}2TiO(HPO_{4})\frac{-H_{2}O}{280-700^{\circ }C}\\ Ti_{2}O{\left(PO_{4}\right)}_{2} \end{array}$$

**Figure 4:**
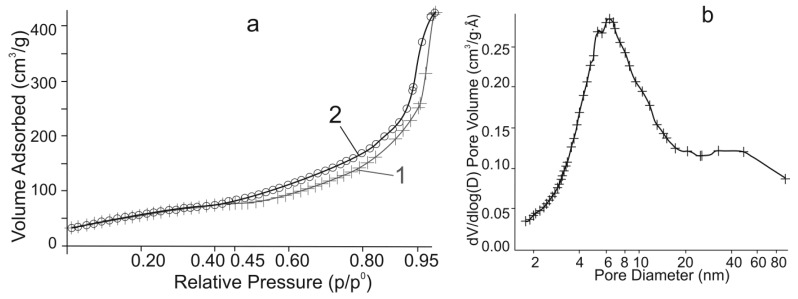
(**a**) N_2_ adsorption (1) and desorption (2), and (**b**) Barrett-Joyner-Halenda (BJH) desorption pore size distribution of titanium phosphate.

From the elemental analysis, XRD, and thermogravimetric and differential scanning calorimetric (TG-DSC) data, the formula of the synthesized product was identified as TiO(OH)H_2_PO_4_·2H_2_O. The synthesized product showed a surface area of 127.9 m^2^∙g^−1^, a total pore volume of 0.26 cm^3^∙g^−1^, and an average pore diameter of 7.2 nm. It can be seen that the pore size distribution was rather broad with a peak of around 6 nm, implying that the material mostly consisted of mesopores (Figure 4).

**Figure 5:**
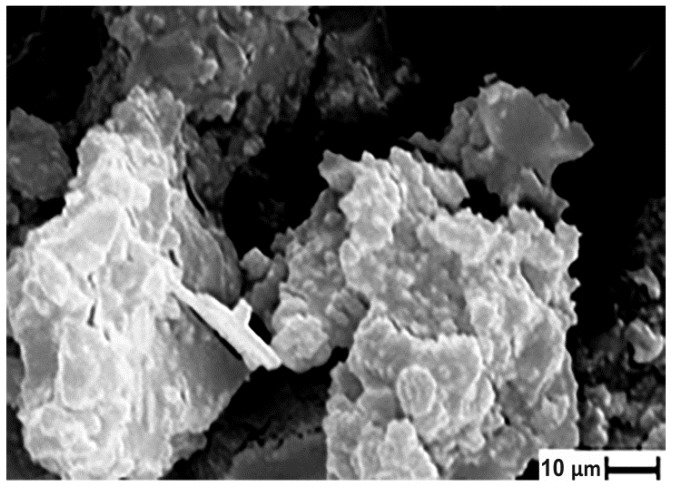
SEM image of the synthesized titanium phosphate.

The morphological features of the material are shown in Figure 5. The obtained solid consisted of agglomerates with an average size of 5–7 µm formed by relatively small particles oriented in one direction.

### 2.2. Chemical Stability Test

**Figure 6:**
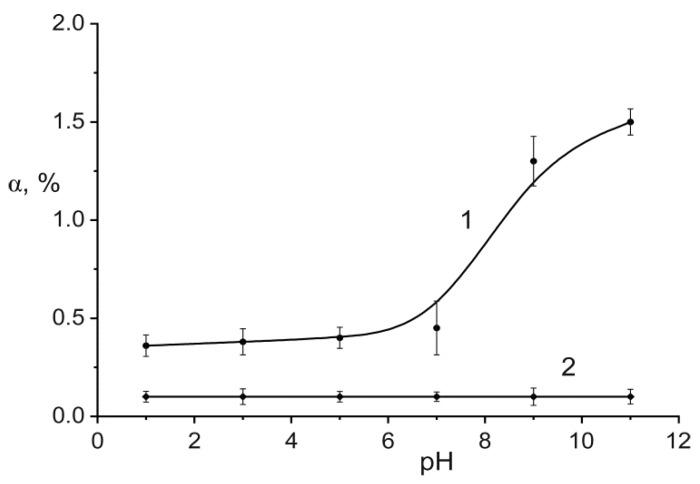
Effect of pH on the degree of titanium and phosphorus leaching (S, %) from the solid into aqueous solutions; 1—P_2_O_5_, 2—TiO_2_ (contact time—10 days, solid (g)-to-liquid (mL) ratio of 1:50).

The chemical stability of obtained material was investigated in a wide pH range from 1 to 11 (Figure 6). Destruction of the sorbent occurred with its hydrolysis. We denoted the degree of Ti and P leaching as an S (Equation (9)). It can be seen that, under chosen conditions, the sorbent had great stability in acid media. With pH increasing, the gradual replacement of the phosphate groups by hydroxyl groups occurred, and the leaching degree in basic media did not exceed 1.5%. With regard to titanium, the low solubility of the hydroxo titanium species provided negligible leaching into solution. The procedure was repeated twice, and the results were averaged. A similar result was reported for titanium phosphate where the maximum leaching of P-species was 2.5% in basic media [26].

### 2.3. Sorption Kinetics and Mechanism of Pb^2+^ and Zn^2+^ Uptake on Titanium Phosphate

To avoid pH changes during the sorption process, Na-substituted titanium phosphate (Na-TiHP) was used. The chemical composition of obtained material is gathered in Table 1. The chemical analysis showed that the molar ratio TiO_2_:P_2_O_5_ was unchanged (1:0.5). This confirms that, under chosen conditions of conversion of H-form into Na-form (pH = 8), the hydrolysis of titanium phosphate did not occur. The sodium uptake was found to be 9.75% or 5.38 meq·g^−1^. This Na^+^ uptake made up 56% of the theoretical ion-exchange capacity (9.6 meq·g^−1^) calculated on the basis of the TiHP formula.

The two protons from the –H_2_PO_4_ groups can be exchanged with Na^+^ ions according to the following reactions:

TiO(OH)(H_2_PO_4_) + Na^+^ ↔ TiO(OH)(HNaPO_4_); (1)

TiO(OH)(HNaPO_4_) + Na^+^ ↔ TiO(OH)(Na_2_PO_4_). (2)

A comparison between the ^31^P-chemical shifts of H- and Na-forms of TiP shows (Figure 1) that the downfield chemical shift changed to 1.7 ppm because of the sodium electronegativity. It is not practical to determine the substituted salt forms due to the amorphous nature of titanium phosphates. From chemical analysis and ^31^P-NMR data, we can suppose that Na-TiHP may be composed of at least two TiP phases: TiO(OH)(HNaPO_4_) and TiO(OH)(Na_2_PO_4_).

**Figure 7:**
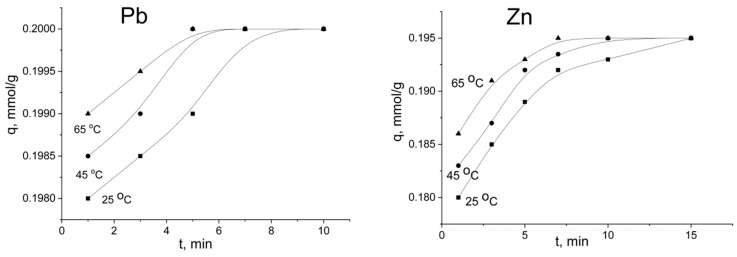
Sorption kinetics of lead and zinc ions at 25–65 °C on titanium phosphate. The initial concentration of Pb^2+^ and Zn^2+^ ions was 1 mmol·L^−1^.

To elucidate the rate-controlling step, kinetic sorption curves were built (Figure 7). According to the results obtained, at low initial concentrations of lead and zinc in the solution (1 mM), the adsorption equilibrium was reached at 5–15 min, dependent on the solution temperature. The metal removal efficiency after 3 min was estimated to be 90–95%. Maximum metal uptake reached 0.2 and 0.195 mmol·g^−1^ for Pb^2+^ and Zn^2+^, respectively. Trublet et al. [26] also showed that the kinetic process on TiO(OH)H_2_PO_4_·H_2_O is relatively fast, and the equilibrium was reached within 10–20 min.

**Figure 8:**
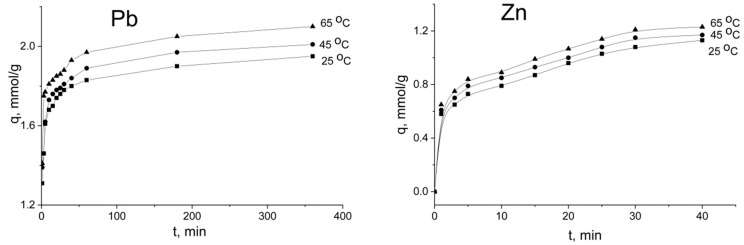
Sorption kinetics of lead and zinc ions at 25–65 °C on titanium phosphate (the initial concentration of Pb^2+^ and Zn^2+^ ions was 10 mmol·L^−1^).

For the 10 mM solution, the metal uptake on the sorbent for 10 min was estimated to be 80–90%, dependent on temperature. Then, the sorption rate started decreasing gradually, and it became almost constant, approaching the equilibrium (Figure 8). At the beginning of the process (the first 5–10 min), the kinetic curves of Pb^2+^ and Zn^2+^ sorption were represented as straight lines. Upon increasing the contact time, the curves started bending. According to the data reported [27], this result indicates that film and intraparticle diffusion affected the rate of the sorption process.

Equilibrium in the system was reached for 30–360 min, depending on the temperature of the solution. At 65 °C, the sorption equilibrium was achieved for 30 min for zinc ions. The kinetics of lead ions was slower; equilibrium was attained for 120 min at the same temperature. However, the sorption capacity (*q*_e_) of the lead ions (2.07 mmol·g^−1^) was higher than that of zinc (1.2 mmol·g^−1^).

In order to determine the mechanism of this process, the Na^+^ release*/*Me^2+^ adsorption for all metal ions was tested (Table 2).

The results show that about 2 mol of Na^+^ ions per 1 mol of metal ion adsorbed was released. Thus, the synthesized titanium phosphate qualified as an ion-exchange material. From the ratio Na^+^/Me^2+^, it can be concluded that no hydrolysis of Na-TiHP occurred. This can be caused by the change in electron density of Na-TiHP functional phosphoric groups. Substituting the protons of the functional groups for sodium ions having an electronegativity lower than that of hydrogen ions led to a redistribution of the electron density by the (*p*–*d*)-conjugation mechanism. At the same time, the *dπ*–*pπ* interaction between the titanium(IV) and the oxygen atoms of the phosphate group was enhanced. The bond between the central atom and the ligand became stronger. As a result, the positive charge on the titanium atom decreased, which made it less susceptible to nucleophilic attack. All these factors led to an increase in the hydrolytic stability of the sample.

According to ion exchange, an equivalent amount of sodium ions was released into the solution from the sodium-substituted form of titanium phosphate during the sorption of lead and zinc. The ion-exchange reaction can be represented as follows:

2Na^+^_s_ + Me^2+^_aq_ ↔ Me^2+^_s_ + 2Na^+^_aq_ (3)

where Na^+^_s_ and Me^2+^_s_ are the sodium and the sorbate ions (Pb^2+^, Zn^2+^) in the sorbent, and Me^2+^_aq_ and Na^+^_aq_ are the sodium and the sorbate ions (Pb^2+^, Zn^2+^) in the solution.

### 2.4. Diffusion Sorption Kinetics of Pb^2+^ and Zn^2+^ Ions

**Figure 9:**
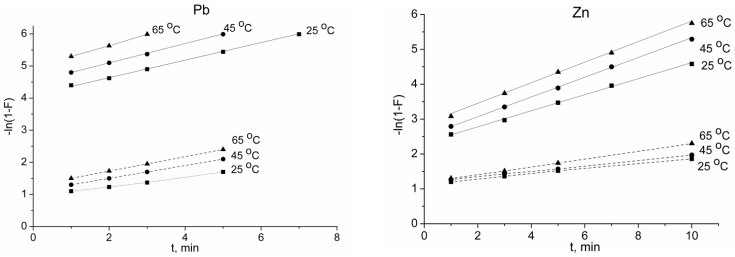
Film diffusion kinetics plots for the lead and zinc sorption on titanium phosphate. The initial concentrations of Pb^2+^ and Zn^2+^ ions were 1 mmol·L^−1^ (solid line) and 10 mmol·L^−1^ (dashed line).

To distinguish between external and internal diffusion, Boyd’s film-diffusion model was applied for the lead and zinc sorption. The plots of ln(1 − *F*) versus *t* fitted to the experimental data are shown in Figure 9. It was established that, for the first 5–10 min, straight lines could be obtained by plotting the experimental data on −ln(1 − *F*) versus *t*. Then, at *F* > 0.8–0.95, the linearity of deviation could be seen at all studied temperatures. The external diffusion rate constant (*k*) could be determined from the slope of the line (Table 3).

The graphs were represented as straight lines that did not intercept the origin. The absence of such an intercept may be due to the influence of internal diffusion processes. Thus, diffusion in the pores of the sorbent particles was also involved in the sorption process at the initial stage.

**Figure 10:**
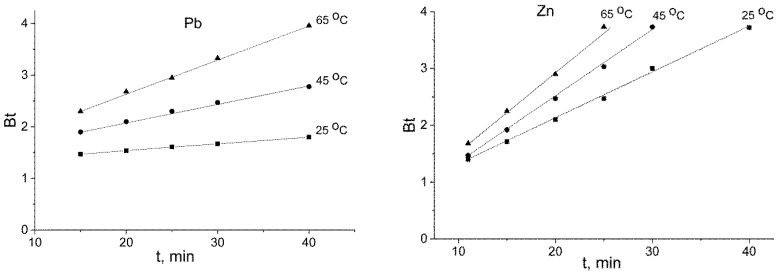
Boyd’s plots for lead and zinc sorption on titanium phosphate at 25–65 °C (the initial concentration of Pb^2+^ and Zn^2+^ was 10 mmol·L^−1^).

For the 10 mM solution, concentration could affect the diffusion processes. To describe the sorption kinetics while taking into account the internal diffusion mechanism, Boyd’s model of gel diffusion was used. Following this model, the plot of *Bt* (calculated using Equation (12)) against time produced a straight line that passed through the origin. Boyd’s coefficient *B* could be determined from the slope of the line, and the effective diffusion coefficient *D_i_* could be calculated according to Equation (11). On the basis of the abovementioned data, external diffusion occurred for the first 5–10 min; thus, the internal diffusion stage was considered to last for the remaining time. The graphs were represented as straight lines that did not intercept the origin (Figure 10). The calculated rate constants of intraparticle diffusion *B* and effective diffusion coefficients *D_i_* are displayed in Table 4. The calculated effective diffusion coefficients *D_i_* were kinetic coefficients that took into account the diffusion transport of the solute in the sorption system. As the lines did not intercept the origin, an error in estimating *D_i_* took place [28]. For the lead sorption on titanium phosphate, the diffusion resistance in the pore volume was much higher than that for zinc.

### 2.5. Adsorption Reaction Models

In order to identify the contribution of chemical interactions to the overall rate of the process, the pseudo-first-order, pseudo-second-order, and Elovich models were applied.

**Figure A1:**
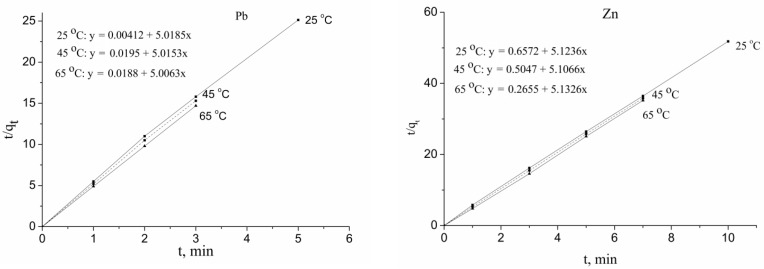
Pseudo-second-order kinetics for Pb^2+^ and Zn^2+^ ion adsorption on titanium phosphate (the initial concentration of metal ions was 1 mmol·L^−1^).

For the 1 mM solution, the plot of the pseudo-first-order equation gave a linear relationship with *R^2^* > 0.97 (Table 5). However, the values of the sorption capacity *q*_e_ calculated by this model were not in agreement with those obtained from the experimental points, *q*_exp_. At the same time, the experimental data correlated well with a linear plot of *t*/*q_t_* versus *t* for the pseudo-second-order reaction model (Figure A1, Appendix A).

For the 10 mM solution, the pseudo-first-order model was also tested. The data corresponding to this model are given in Table 6. It can be seen that the sorption capacities differed from the experimental data, which indicated that the reaction was unlikely a first-order reaction.

**Figure A2:**
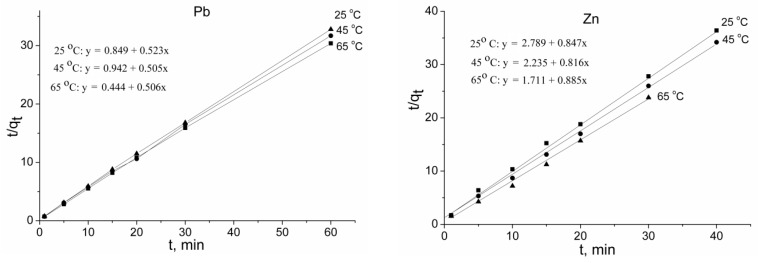
Pseudo-second-order kinetics plots for Pb^2+^ and Zn^2+^ ion adsorption on titanium phosphate (the initial concentration of metal ions was 10 mmol·L^−1^).

Better results were obtained by using the pseudo-second-order model; all regression coefficients (*R^2^*) were close to 1 (Figure A2, Appendix A; Table 6). The values of the sorption capacity calculated by this model were in very good agreement with the experimental data.

**Figure A3:**
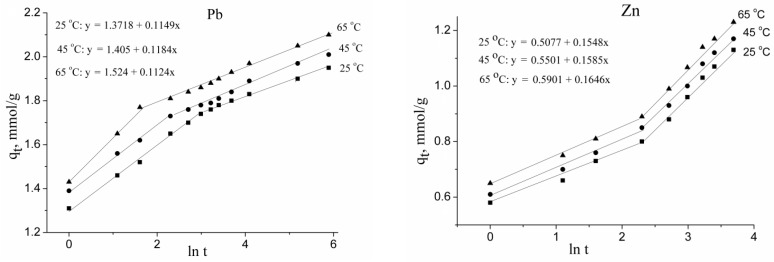
Elovich kinetics plots for Pb^2+^ and Zn^2+^ ion adsorption on titanium phosphate (the initial concentration of metal ions was 10 mmol·L^−1^).

The analysis of the experimental data by the Elovich model showed that the kinetic curves demonstrated two slopes (Figure A3, Appendix A). The presence of separate linear areas indicated a change in the energy of the interaction between the solute and the sorbent during the sorption process. The initial sorption rate constant of lead ions *α* was much higher than the desorption constant *β*, which indicated a high affinity of Pb^2+^ to this sorbent. These constants were comparable with their values for Zn^2+^ ions (Table 6).

### 2.6. Selectivity Test

In this research, titanium phosphate was tested for wastewater purification. A model solution having the composition of real mine water before liming (Pb^2+^—50 mg·L^−1^, Zn^2+^—60 mg·L^−1^) was prepared. The effect of the pH values of wastewater (2.5–4.5) on the sorption capacity of the sorbent was investigated. According to the data obtained, the sorption of lead and zinc ions did not depend on the pH of the solution. The removal efficiency of heavy metal ions was more than 99.9% at a pH ranging from 2.5 to 4.5 after 10 min of sorption. This phenomenon was related to a lower concentration of the solute compared to the number of active surface sites.

The solution purification after neutralization was also tested. After the chemical treatment, the concentrations of metals in the model solution were 5 and 10 mg·L^−1^ for Pb^2+^ and Zn^2+^, respectively. The concentration of calcium ions was 0.48 g·L^−1^; the pH value was 5.5.

In order to investigate the sorbent selectivity toward the Pb^2+^ and Zn^2+^ ions, the distribution coefficient *K*_d_ (Table 7) was determined according to the Equation (21).

Table 7 shows that the distribution coefficient in the presence of calcium was more than 10^5^ mL·g^−1^ for all chosen concentrations. The *K*_d_ values for Ca^2+^ in the presence of heavy metals did not exceed 23 mL·g^−1^. The separation coefficient *β* = *K*_d(Pb, Zn)_/*K*_d(Ca)_ for the studied condition was found to be in the range 0.89–3.6 × 10^4^ for *K*_d(Pb)_/*K*_d(Ca)_ and 0.56–2.4 × 10^4^ for *K*_d(Zn)_/*K*_d(Ca)_.

## 3. Discussion

There are five stages of ion exchange during which the solute is adsorbed. For the process to begin, the solute must be delivered to the sorbent surface from the solution. This stage (the first one) depends on the value of the solute diffusion coefficient (D, cm^2^·s^−1^) in the external solution. The next stage involves overcoming the interface solution (thin liquid film on the surface of the sorbent particles), and the solute transition through this film to the solid surface. The third stage is the diffusion of the solute in the pores and/or along the pore walls. This stage depends on the properties of the solute (size, charge value, hydratability) and the properties of the sorbent (degree of the ionization of functional groups, pore size, etc.). The fourth stage relates to the ion-exchange process. Then, it takes some time to remove the displaced ion from the solid phase. In other words, it takes some time to diffuse the counter-ion into the external solution through a liquid film of the sorbent surface (fifth stage). From the given series of the sorption process, the ion-exchange reaction rate is determined by the slowest stage [21].

Investigation of the ion-exchange kinetics is of great importance because it develops an understanding of the controlling reaction step and the sorption mechanisms.

As can be seen from Figure 7 and Figure 8, the initial concentration of the solute affected the sorption equilibrium. For the 1 mM solution, the adsorption equilibrium was reached for 5–15 min depending on the solution temperature. The large difference in concentration between the active surface sites and the solutes in the boundary layer provided fast sorption kinetics. For the 10 mM solution, when the solute concentration was higher than the sorption capacity of TiP, no significant increase in adsorption was observed after 30 min for zinc and after 180 min for lead ions. The adsorption capacity of the sorbent was increased dramatically at the beginning of the process, and metal uptake on the sorbent was estimated to be 80–90%. Jia et al. [22] found that the kinetics of lead and zinc ions on powder TiP reached equilibrium within 1 h at the initial concentration of the target ions of 0.5 mM·L^−1^. For a granulated material, a contact time of 300 min is required to achieve the sorption equilibrium. The obtained sorption capacity (2.07 mmol·g^−1^ for lead ions and 1.2 mmol·g^−1^ for zinc) was higher compared with known TiP values. Clearfield et al. found that titanium phosphate with –HPO_4_^2^^−^ and –H_2_PO_4_^−^ groups exhibits a sorption capacity toward lead and zinc ions up to 1.2 mmol·g^−1^ and 0.3 mmol·g^−1^, respectively [29]. For amorphous titanium phosphate with a general formula of Ti(HPO_4_)_2_·H_2_O, the sorption capacity was found to be 1.4 mmol·g^−1^ for Pb^2+^ and 0.52 mmol·g^−1^ for Zn^2+^ ions [30].

The difference between the sorption capacities of zinc and lead ions relates to the difference in the size of their hydrated shells. The crystal ionic radius of Pb^2+^ (126 pm) is greater than that of Zn^2+^ (80 pm) [31]; thus, conversely, the radius of hydrated shells of Pb^2+^ is smaller.

To calculate the effective radius of the adsorbed ion (*r*_s_), the size of the available site of solid (*S*) is estimated. For the calculation of *S*, we assume that the entire surface is available for the sorption of ions,

*S* = *S*_BET_/(*q*·*N*_a_), (4)

where *S*_BET_ is the surface area determined by the Brunauer-Emmett-Teller (BET) method, *q* is the maximum monolayer coverage capacity (mmol·g^−1^) determined from the Langmuir isotherm, and *N*_a_ is Avogadro’s constant.

The effective radius of the adsorbed ion proposed was calculated as follows:

*r*_s_ = (1/2)(*S*)^1/2^ (5)

Stokes radius was calculated according to the following equation:

*r*_aq_ = *zF*^2^/(6π*N_a_ηλ*°) (6)

where *z* is the charge of the metal ion, *F* is the Faraday constant, *η* is the viscosity of water at 25 °C, and *λ*° is equivalent conductivity of ion in an aqueous solution at 25 °C.

The calculated values of the Stokes radius were 262 pm and 346 pm, and the effective radii of adsorbed ions were 156 pm and 209 pm for Pb^2+^ and Zn^2+^ ion, respectively. The greater effective radius of Zn^2+^ ion caused a lower sorption capacity compared to Pb^2+^ ion due to the fact that the adsorbed Zn^2+^ ions occupied a larger area on the surface of the sorbent.

The effective radii of the adsorbed ions were smaller than their Stokes radii and larger than their crystal ionic radii.

It is obvious that the sorption of the ions was accompanied by dehydration of their shells. The dehydration degree (*α*, %) of adsorbed ions can be estimated as follows:

(7) $$\alpha \;=\;\frac{4/3\pi \left(r_{aq}^{3}-r_{s}^{3}\right)}{4/3\pi \left(r_{aq}^{3}-r_{cr}^{3}\right)}\;=\;\frac{r_{aq}^{3}-r_{s}^{3}}{r_{aq}^{3}-r_{cr}^{3}}$$

where *r*_aq_ is the Stokes radius, *r*_cr_ is the crystal ionic radius, and *r*_s_ is the radius of adsorbed ion.

The calculated values of the shell dehydration degree at 25 °C were 88.7% and 78.9% for Pb^2+^ and Zn^2+^ ions, respectively. The experimental data showed that a higher degree of dehydration of lead ions corresponded to slower kinetics.

The film and intraparticle diffusion kinetics plots for the lead and zinc sorption on titanium phosphate showed that the graphs were represented as straight lines that did not intercept the origin, indicating that more than one step took place in the adsorption processes. The obtained results could be attributed to the sorption stages of the external and internal surfaces and intraparticle diffusion. An increase in the temperature of the solution led to an increase in the external diffusion rate. The close values of the rate constants and the similar energy consumption for Pb^2+^ and Zn^2+^ indicated that there was no significant effect of the size of the ions on the diffusion at this stage for the 1 mM solution.

The reduced values of the internal diffusion rate for the concentrated solution (10 mM) could have been caused by a sharp increase in the ions flow through the film. This, in turn, caused steric or electrostatic hindrances for hydrated ions which were to be adsorbed. The steric hindrances for Zn^2+^ were greater than those for Pb^2+^ due to the greater hydrated shell of zinc. This was confirmed by the slower external diffusion rate for Zn^2+^ compared to Pb^2+^. The values of the activation energy at this stage for the 10 mM solution were higher compared to those for the 1 mM solution.

To assess the difficulty of ion mass transfer in the pores, the values of the self-diffusion coefficients of Pb^2+^ and Zn^2+^ions in the water at 25 °C were calculated according to the following formula [32]:

*D_i_*^o^ = (*RTλ*)/(*F*^2^*z*) (8)

where *R* is the gas constant, *T* is the temperature (K), *λ* is the limiting electrical conductivity of the solute, *F* is Faraday’s number, and *z* is the charge of the ion.

The calculated self-diffusion coefficients for lead and zinc were 9.3 × 10^−10^ and 7.13 × 10^−10^ m^2^∙s^−1^, respectively. These values compared to those for the effective diffusion coefficients (Table 4) confirmed that pore diffusion resistance took place for the lead and zinc sorption.

The values of the activation energy were found to be 32.62 (*R^2^* = 0.981) and 12.11 kJ·mol^−1^ (*R^2^* = 0.993) for lead and zinc, respectively. Values of the activation energy below 80 kJ·mol^−1^ are quite typical for the diffusion of ions in sorbent pores [33]. It should be noted that the calculated B and *D_i_* values for Zn^2+^ were higher than those for Pb^2+^. We expected that the diffusion rate would be higher for Pb^2+^ due to its smaller hydrated shell. The obtained results could be explained both by a stronger ion–ion interaction in the pore volume and by the affinity of Pb^2+^ to titanium phosphate compared to Zn^2+^. The strong interaction between lead and the sorbent surface impeded the diffusion of the ions in the pore volume. These interactions could cause higher values of the activation energies for lead than for zinc.

To describe the sorption kinetics taking into account the chemical interaction, the pseudo-first-order, pseudo-second-order, and Elovich models were applied.

The pseudo-first-order equation properly describes the sorption characteristics if film diffusion has a marked effect on the process. The pseudo-second-order equation allows taking into account the sorbate–sorbent interactions, as well as the intermolecular interactions of adsorbed species. According to the Elovich model, the process is chemical adsorption on an energetically heterogeneous surface, and both sorption and desorption processes influence the kinetics of the solute uptake. It should be noted that desorption processes have a considerable impact when approaching the equilibrium.

For all studied ions, better results were obtained by using the pseudo-second-order model. The calculated values of *q*_e_ coincided with the values of *q*_exp_. For the 1 mM solution, the calculated value of *k*_2_ was in good agreement with the value of *k*_2_ obtained by Trublet [26]. The high *k*_2_ values explained the rapid (within the first minutes) almost complete sorption of Pb^2+^ and Zn^2+^ ions. The calculated values of the activation energy and high values of the rate constant indicated a free exchange of ions and low energy consumption on the partial dehydration of the sorbed ion. The lower *k*_2_ values for Zn^2+^ may have been due to its larger hydration shell, which prevented the sorbed ion from interacting with the sorbent surface. The obtained results verified that the radius of the ion and the size of its hydrated shell affect the sorption process. For the 10 mM solution, a sharp decrease in the values of the rate constants compared to the values obtained for the 1 mmol·L^−1^ solution could have been due to the strong influence of the sorbate–sorbate interaction on the surface of titanium phosphate, with a high loading degree of the sorbent with metal ions. The effect of this interaction was greater for lead sorption.

It should be noted that, with a small sorbent loading (sorption in the 1 mM solution), when the interaction between sorbed metal ions did not have a significant effect on the sorption process, the values of the diffusion rate constant and the constant of the metal ion interaction with the sorbent, according to the pseudo-second-order reaction, were large (Table 5). These values became significantly lower when sorption took place in the more concentrated solution (10 mM) (Table 6). In this case, sorption was accompanied by a high sorbent loading, approaching the maximum sorption capacity. A strong decrease in the values of the diffusion rate and pseudo-second-order reaction constants was caused by an increase in interferences during mass transfer in the pores, as well as during the interaction between the sorbate and sorbent. It is obvious that diffusion had a significant effect on the overall rate of the sorption process.

The obtained value of *k*_2_ (0.0015 g·mg^−1^·min^−1^) for lead was much greater than that obtained by Kapnisti et al. [23] for titanium phosphate with Ti_2_O_3_(H_2_PO_4_)_2_ composition (0.000227g·mg^−1^·min^−1^). This difference may be due to a lower initial lead concentration in the solution, as well as better pore characteristics of the synthesized sorbent (0.05 cm^3^/g for Ti_2_O_3_(H_2_PO_4_)_2_ and 0.26 cm^3^/g for the studied sorbent).

For the Elovich model, the initial sorption rate constant of lead ions *α* was much higher than the desorption constant *β*, which confirmed a high affinity of Pb^2+^ to this sorbent. These constants were comparable for Zn^2+^ ions.

**Figure A4:**
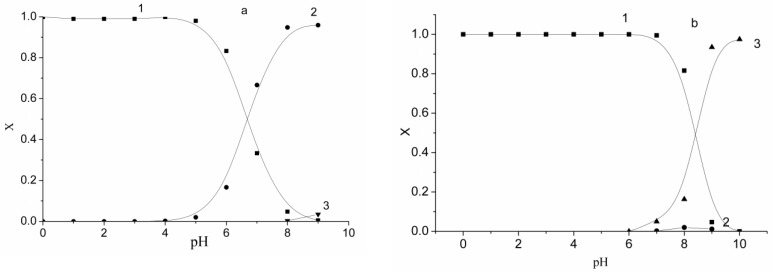
The distribution diagram for chemical species of lead(II) and zinc(II) in the solution as a function of pH (**a**): 1—Pb^2+^, 2—PbOH^+^, 3—Pb(OH)_2_; (**b**): 1—Zn^2+^, 2—ZnOH^+^, 3—Zn(OH)_2_.

The selectivity of TiP toward lead ions was confirmed using real mine water purification (Table 7). Neutralization of acidic lead–zinc mine water is currently used before discharging the water into open-water bodies. Traditionally, calcium hydroxide or calcium carbonate is applied for this purpose. When neutralizing the solution to a pH of 6–7, toxic metals remain in the wastewater as a hydrolyzed species (see Figure A4, Appendix A). This leads to the pollution of water bodies. A further increase in pH to 8.5–9 results in the precipitation of metal cations as hydroxides; however, even in this case, the residual zinc and lead concentrations are 5–10 mg·g^−1^, which is higher than the TLV. The investigation shows that the removal efficiency of lead and zinc ions was more than 99.9% and did not depend on the chosen pH range of the solution. Titanium phosphate demonstrated high selectivity toward the studied metal ions, and there was no competitive effect of the other cations observed. The distribution coefficients *K*_d_ for Pb^2+^ and Zn^2+^ ions were found to be 10^5^ in the presence of Ca^2+^ ions. The residual concentration of toxic metals in the solution did not exceed 0.01 mg·L^−1^, i.e., lower than the TLV.

The high chemical affinity of studied metal ions to phosphate groups makes the basis for the application of titanium phosphate as a promising material for immobilization of lead and zinc spices into the phosphate matrix.

## 4. Materials and Methods

### 4.1. Synthesis of Titanium Phosphate

Titanium salt, (NH_4_)_2_TiO(SO_4_)_2_·H_2_O, was used to synthesize titanium phosphate. Firstly, 50 g of salt was gradually added to a 30% H_3_PO_4_ solution so that the molar ratio was TiO_2_:P_2_O_5_ = 1:1.5 according to the synthesis procedure by Maslova [19]. The suspension was further stirred for 4 h at 60 °C and then filtered. To remove the ammonium cations, which are represented as ammonium phosphate groups in titanium phosphate, the obtained precipitate was firstly washed with 0.1 N HCl and then with deionized water. The resulting solid was dried at 60 °C.

### 4.2. Characterization Techniques

Elemental analyses were carried out by dissolving the sorbent in a mixture of HF, HNO_3_, and HCl, and the solutions were analyzed by direct-current plasma emission spectroscopy using a Shimadzu ICPE-9000 spectrometer (Shimadzu Corporation, Tokio, Japan). The ^31^P-MAS NMR spectra were obtained on a Bruker Avance III 400 MHz spectrometer (Bruker AG, Zurich, Switzerland). All data were reported with chemical shifts related to H_3_PO_4_ at 0 ppm, which was used to investigate the samples. The thermogravimetric (TG/DTG) and differential scanning calorimetric (DSC) data of the sample were collected using a thermogravimetric analyzer Netzsch STA 409 PC/PG (NETZSCH-Geratebau GmbH, Selb, Germany) under argon atmosphere. Powder XRD data were obtained on a Shimadzu D6000 (Shimadzu Corporation, Tokio, Japan) diffractometer with monochrome *CuK_α_* radiation (λ = 1.5418 Å). The surface area of the samples was determined by low-temperature nitrogen adsorption, using a surface analyzer Tristar 320 (Micromeritics Company, Norcross, Georgia, USA). The pore size distribution was calculated using the BJH method. The concentration of heavy metals in the filtrates from all sorption experiments was determined by atomic adsorption on an AAS 300 Perkin-Elmer (PerkinElmer Inc., Waltham, Massachusetts, USA) spectrometer. The sieve size analysis was carried out to estimate the average size of sorbent particles. A fraction of 0.1 mm particle size was selected for the study.

Deionized water was used in all experiments. H_3_PO_4_, HCl, NaOH, and the metal salts (Pb(NO_3_)_2_, Zn(NO_3_)_2_·6H_2_O, and Na_2_CO_3_) were purchased from Neva-Reaktiv (Saint-Petersburg, Russia). All chemicals were of analytical reagent grade and were used without further purification.

### 4.3. Chemical Stability Test

The chemical stability test was carried out in batch experiments in ambient conditions. Firstly, 1 g of titanium phosphate was mixed with 50 mL of solution, and the suspension was kept in closed vessels under constant stirring for 10 days. The pH of the solutions was adjusted by adding 1 M HCl or NaOH. The Ti and P amounts in the filtrates were determined by inductively coupled plasma atomic emission spectrometry (ICP-AES), and the degree of titanium and phosphorus leaching from the solid into aqueous solutions (**S**, %) was calculated using the following expression:

**S** = (*CV*)/(*m*/*M*) (9)

where *C* is the equilibrium concentration of Ti or P in the solution after 10 days (mg·L^−1^), *V* is the solution volume (L), *m* is the mass of the sorbent (mg), and *M* is the molar mass of the sorbent (mg·mmol^−1^).

### 4.4. Sorption Experiment and Conditions

All solutions were prepared by dissolving initial salts in deionized water. The concentration range for the sorption experiments was selected based on the solubility of the sorbate and the composition of the formed complexes. It is well known that heavy metal cations show a strong tendency to hydrolysis, with the formation of insoluble hydroxides. Therefore, for the correct analysis of the adequacy of the experimental data obtained for the considered sorption models, it was important to select conditions under which the sorbate was soluble and existed in solution in one dominant form.

The concentrations of solute can affect the sorption kinetics; thus, 1 and 10 mmol∙L^−1^ solutions were selected for the sorption experiments. The selection of these experimental conditions was based on the distribution of the complexes of lead(II) and zinc(II) formed depending on the pH of the solution [32] (Figure A4, Appendix A). According to the diagram obtained, Pb^2+^ ions were present in the solution as the dominant form of lead(II) at pH < 5. Zn^2+^ ions were present as the dominant form of Zn(II) in the solution at pH < 7.

At the concentrations of Pb(II) and Zn(II) of 1 mmol∙L^−1^, the pH values of the solutions were 4.6 and 6.3, respectively. At the concentrations of Pb(II) and Zn(II) of 10 mmol∙L^−1^, the pH values were 4.1 and 5.8, respectively. At these pH values, the degree of hydrolysis of metal ions is negligible. The bulk of metal ions existed in the form of divalent ions, Pb^2+^ and Zn^2+^.

For sorption experiments, the obtained titanium phosphate was treated with a 0.1 M solution of sodium carbonate at a mass (g)/volume (mL) ratio = 1:200. The suspension was kept under stirring in ambient conditions for 24 h before filtration. The resulting solid was washed with water until the pH was ~6. The Na-substituted titanium phosphate obtained was used in this study.

The sorption kinetics of lead and zinc cations from aqueous solutions of metal nitrate were studied at 25 °C, 45 °C, and 65 °C. The batch technique was employed for these purposes. The initial Pb^2+^ and Zn^2+^ concentrations in the solution ranged from 10^−2^ to 10^−3^ M. The sorbent (1 g) was mixed with 200 mL of corresponding solutions (pH = 7) under vigorous stirring (300 rpm) in all experiments. The initial solution was agitated in a thermostat for 1 h at the desired temperature, after which the sorbent was added. The concentration of the metals at all points within the bulk of the solution and on the sorbent surface was assumed to be constant at a given rotation speed, i.e., diffusion in a well-stirred solution was considered. This implies a high rate of sorbate transport from the bulk solution to the sorbent surface. To establish the amount of maximum metal uptake, the suspensions were stirred for 24 h to ensure that equilibrium was reached. The concentrations of the metal in the solution before and after the sorption processes were determined. The pH control was carried out during the sorption experiments. It was found that the pH of the filtrates remained in the same range as the starting pH.

### 4.5. Kinetic Models

For porous sorbents, the adsorption rate can be controlled by external film mass transport (film diffusion) or mass transfer of solutes within the particle (diffusion in the pores or migration along the pore surface), i.e., internal or intraparticle diffusion [28].

Boyd’s diffusion, Lagergren pseudo-first-order, Ho and McKay pseudo-second-order, and Elovich models were applied for modeling sorption kinetics.

Boyd’s diffusion model is one of the most widely used models for the investigation of adsorption mechanisms [34,35]. The analysis of the kinetic data allows establishing the rate-limiting step (film diffusion or intraparticle diffusion) and determining an effective diffusion coefficient [36].

(10) $$F\;=\;1\;-\;\frac{6}{\pi^{2}}\sum_{n=1}^{\infty }(1/n^{2})\exp\left(-\frac{D_{i}t\pi^{2}n^{2}}{r^{2}}\right)$$

where *D*_i_*π*^2^/*r*^2^ = *B* is the kinetic coefficient, *F* is the fractional attainment at equilibrium, *r* is the average radius of the sorbent particles (m), *D_i_* is the effective diffusion coefficient (m^2^·s^−1^), *t* is the sorption time (sec), and *n* represents integers from 1 to infinity.

The fractional attainment of equilibrium was calculated by the formula *F* = *q*_t_/*q*_e_, where *q*_t_ is the amount of the sorbate at any time *t* (mmol·g^−1^), and *q*_e_ is the amount of the sorbate at equilibrium (mmol·g^−1^).

Effective diffusion coefficients were calculated using the following expression [37]:

(11) $$D_{i}\;=\;\frac{r^{2\;}}{\pi^{2}}B$$

where *r* is the average radius of the sorbent particles (m).

The interrelation between the fractional attainment at equilibrium *F* and the kinetic coefficient *B* could be calculated by the following Equation [38]:

*Bt* = −2*F*·lg(1 − *F*) (12)

In dilute solutions, film diffusion can be the rate-limiting step, and its equation can be expressed as follows [39]:

−ln(1 − *F*) = 3(*D_i_ct*)/(*rδm*) (13)

where *D_i_* is the coefficient of the substance diffusion through a film of thickness *δ*, covering the sorbent grain, *r* is the sorbent particle radius, *t* is the contact time, and *c* and *m* are the concentrations of the sorbate and sorbent in the solution, respectively.

The temperature dependence of the rate constant is described by the Arrhenius equation [40].

*k* = A·exp(−*E*_a_/*RT*) (14)

This equation can be rearranged into the logarithmic form as follows:

ln*k* = lnA − *E*_a_/*RT* (15)

where *E*_a_ is the activation energy, *R* is the gas constant, *T* is the absolute temperature, and A is the Arrhenius pre-exponential factor. By plotting ln*k* versus 1/*T*, *E*_a_*/R* can be determined from the slope of the straight line.

For the intraparticle diffusion mechanism, the values of the self-diffusion coefficients of Pb^2+^ and Zn^2+^ ions in water at 25 °C were calculated according to the following formula [32]:

*D_i_*^o^ = (*RTλ*)/(*F*^2^*z*) (16)

where *R* is the gas constant, *T* is the temperature (K), *λ* is the limiting electrical conductivity of solute, *F* is Faraday’s number, and *z* is the charge of the ion.

Using the effective diffusion coefficients, the values of the activation energy of the sorption process at the intraparticle diffusion stage were calculated by an equation similar to the Arrhenius one [41].

*D_i_* = *D*·exp(−*E*_a_/*RT*) (17)

where *E*_a_ is the activation energy, kJ∙mol^−1^, *D* is the constant, m^2^∙s^−1^, *R* is the gas constant, J∙mol^−1^, and *T* is the temperature, K.

In order to identify the contribution of chemical interactions to the overall rate of the process, the pseudo-first-order, pseudo-second-order, and Elovich models were used.

The Lagergren pseudo-first-order model equation may be expressed as *d*q_1_/*d*t = *k*_1_(*q*_e_ − *q*_t_) or as a linear form [42,43].

lg(*q*_e_ − *q*_t_) = lg*q*_e_ − *k*_1_*t*/2.3 (18)

where *q*_e_ and *q*_t_ are the amounts of metal cation sorbed at equilibrium and at time *t* (mmol·g^−1^), respectively, and *k*_1_ is the rate constant (min^−1^).

The plot of lg(*q*_e_ *− q*_t_) versus t allows determining the rate constant of the sorption and amount of metal cation uptake at equilibrium. This equation describes the film diffusion processes which control the adsorption rate for the first few minutes of the sorption in the experiments with stirring [44].

The Ho and McKay pseudo-second-order equation [41] is widely used to describe the kinetic characteristics of adsorption. In a linear form, this equation can be represented as follows [45]:

*t*/*q*_t_ = 1/(*k*_2_·*q*_e_^2^) + *t*/*q*_e_ (19)

where *k*_2_ is the rate constant (g·mmol^−1^·min^−1^).

The plot of *t/q*_t_ against *t* in Equation (8) should give a linear relationship, which allows determining *q*_e_ and *k*_2_ from the slope and intercept of the straight line.

The Elovich exponential model describes the kinetics of heterogeneous chemisorption on solid surfaces [27]. The Elovich equation simplified by Chien and Clayton can be written as follows [46]:

*q*_t_ = (1/*β*)ln(*αβ*) + (1/*β*)ln(*t*) (20)

where *q*_t_ is the sorption capacity at time *t* (mmol·g^−1^), *α* is the initial sorption rate constant (mg·g^−1^·min^−1^), and *β* is the desorption constant (g·mmol^−1^).

The constants *α* and *β* were calculated from the slope and the intercept of the plot of *q*_e_ versus ln(*t*), respectively.

### 4.6. Selectivity Test

For all experiments, the solid-to-liquid ratio (g:mL) was 1:200.

In order to investigate the sorbent selectivity toward the Pb^2+^ and Zn^2+^ ions, the distribution coefficient *K*_d_ (Table 7) was determined according to the following equation:

(21) $$K_{d}=\;\frac{\;C_{o\;}-C_{e}}{C_{e}}\;\cdot \;\frac{V}{m}$$

where *C_o_* is the initial concentration of the metal ion in solution (mmol·L^−1^), *C_e_* is the concentration of the metal ion at equilibrium (mmol·L^−1^), *V* is the volume of the solution (mL), and *m* (g) is the mass of the adsorbent used in the sorption experiments.

## 5. Conclusions

For the first time, the sorption kinetics of Pb^2+^ and Zn^2+^ cations on mesoporous titanium phosphate considering external and internal mass transfer, as well as chemical interactions, were thoroughly studied. The material was synthesized from a new crystalline precursor, (NH_4_)_2_TiO(SO_4_)_2_·H_2_O. The analysis of the results obtained was carried out taking into account the differences in the degree of sorbed ion hydration.

Boyd’s external and internal diffusion models were used to analyze the kinetics of the ion-exchange processes. It was shown that diffusion proceeds according to a mixed mechanism.

The gradient of the concentration can be a driving force for the sorption of the solute from the surface into the pores of the sorbent. An increase in the initial concentration of the solute (10 mM) led to sharp increase in the activation energy and a decrease in the external diffusion rate constant. The values of the rate constant of the pore diffusion and effective diffusion coefficients for Zn^2+^ were significantly higher than those for Pb^2+^. A higher diffusion hindrance in the pores for Pb^2+^ compared to Zn^2+^ could have been caused by a stronger interaction of Pb^2+^ cations, both with each other and with the active surface sites of the sorbent.

For all selected solute concentrations, the chemical interaction was described by a pseudo-second-order reaction. The sorption kinetics was regulated by the effective radius of the hydrated ions. Additionally, synthesized titanium phosphate was tested for removing toxic metal ions from mine water before and after liming. The fast kinetics and the ability to efficiently adsorb the low concentrations of zinc and lead in a wide pH range make this material a very promising sorbent of toxic metal cations. The residual concentration of toxic metals in the solution did not exceed 0.01 mg∙L^−1^, which is five and 200 times lower than the TLV for Pb^2+^ and Zn^2+^, respectively. The heat treatment of the spent sorbent led to the formation of stable crystalline insoluble metal phosphates, which were capable of firm immobilization of toxic cations in the material structure. The investigation of the sorption equilibrium will be the purpose of further studies.

## Figures and Tables

**Table 1:** Elemental analysis data for titanium phosphate products.

Samples	Solid Phase Content (%)				Composition
	TiO_2_	P_2_O_5_	H_2_O	Na_2_O	
TiHP	38.22 ± 0.05	33.77 ± 0.05	28.01 ± 0.05	-	TiO(OH)H_2_PO_4_⋅2H_2_O
Na-TiHP	34.27 ± 0.05	30.45 ± 0.05	20.39 ± 0.05	14.89 ± 0.05	TiO(OH)Na_1.12_H_0.88_PO_4_⋅1.7H_2_O

**Table 2:** Stoichiometry of metal ion exchange on titanium phosphate. The initial concentration of metal ions was 10 mM, with a solid-to-liquid ratio (g:mL) of 1:200.

	Equilibrium Concentration		Ion Exchange		
t, min	Me^2+^, mmol·L^−1^	Na^+^, mmol·L^−1^	Me^2+^ Uptake, mmol	Na^+^ Release, mmol	Me^2+^/Na^+^ Ratio
**Pb**					
1	3.45	13.34	1.31	2.67	1:2.04
3	2.71	14.67	1.46	2.93	1:2.01
5	1.95	16.23	1.61	3.25	1:2.02
10	1.63	16.98	1.67	3.40	1:2.03
15	1.50	17.10	1.70	3.42	1:2.01
20	1.30	17.40	1.74	3.48	1:2.00
25	1.21	17.60	1.64	3.52	1:2.15
30	1.12	17.70	1.78	3.54	1:1.99
40	1.03	18.00	1.79	3.60	1:2.01
60	0.85	18.13	1.83	3.63	1:1.98
180	0.50	18.75	1.90	3.75	1:1.98
360	0.25	19.20	1.95	3.84	1:1.97
**Zn**					
1	7.10	6.12	0.58	1.22	1:2.11
3	6.75	6.67	0.65	1.33	1:2.05
5	6.35	7.38	0.73	1.48	1:2.02
10	6.05	8.05	0.79	1.61	1:2.04
15	5.65	8.72	0.87	1.74	1:2.00
20	5.20	9.57	0.96	1.91	1:1.99
25	4.85	10.31	1.03	2.06	1:2.00
30	4.60	10.68	1.08	2.14	1:1.98
40	4.35	11.17	1.13	2.23	1:1.98

**Table 3:** Kinetic parameters of the film diffusion model for Pb^2+^ and Zn^2+^ sorption at different temperatures and solute concentrations on titanium phosphate.

Parameters	Pb^2+^			Zn^2+^		
*T*, °C	25	45	65	25	45	65
1 mM solution						
*k*, min^−1^	0.263	0.297	0.345	0.228	0.279	0.294
*R^2^*	0.996	0.997	1.0	0.996	0.994	0.997
*E_a_*, kJ∙mol^−1^	5.19 ± 0.43			5.03 ± 0.48		
10 mM solution						
*k*, min^−1^	0.152	0.2	0.225	0.070	0.077	0.111
*R^2^*	0.995	1.0	1.0	0.997	0.999	0.997
*E*_a_, kJ∙mol^−1^	8.10 ± 0.62			8.39 ± 0.77		

**Table 4:** Kinetic parameters of the intraparticle diffusion model for Pb^2+^ and Zn^2+^ ion sorption at different temperatures on titanium phosphate (the initial concentration of Pb^2+^ and Zn^2+^ was 10 mM·L^−1^).

	Pb^2+^			Zn^2+^		
*T*, °C	25	45	65	25	45	65
*D_i_*·10^−10^, m^2^·s^−1^	0.14	0.35	0.67	0.82	1.18	1.47
*B*, min^−1^	0.013	0.035	0.066	0.081	0.117	0.145
*R^2^*	0.999	0.994	0.997	0.989	0.997	0.998
*E*_a_, kJ∙mol^−1^	32.62 ± 0.84			12.11 ± 0.96		

**Table 5:** Kinetic parameters of pseudo-first-order and pseudo-second-order models for sorption of zinc and lead ions on titanium phosphate (the initial concentration of metal ions was 1 mmol·L^−1^).

Kinetic Model	Parameters	Pb^2+^			Zn^2+^		
		Temperature, K					
		298	318	338	298	318	338
Pseudo-first order	*q*_exp_, mmol∙g^−^^1^	0.199	0.200	0.200	0.194	0.195	0.195
	*k*_1_, min^−^^1^	0.075	0.088	0.150	0.102	0.156	0.163
	*q*_e_, mmol∙g^−^^1^	0.0024	0.0018	0.0014	0.0190	0.0182	0.0129
	*R^2^*	0.997	0.999	0.999	0.989	0.978	0.998
	*E*_a_, kJ∙mol^−^^1^	14.29 ± 0.84			9.94 ± 0.76		
Pseudo-second order	*k*_2_·10^4^, g·mol^−^^1^·min^−^^1^	61	129	133	4.0	5.2	9.9
	*q*_e_, mmol∙g^−^^1^	0.199	0.199	0.200	0.195	0.195	0.195
	*R^2^*	1	1	1	0.999	0.999	1
	*E*_a_, kJ∙mol^−^^1^	16.49 ± 0.52			18.76 ± 0.64		

**Table 6:** Kinetic parameters of the models for lead and zinc ion adsorption on titanium phosphate (the initial concentration of metal ions was 10 mM·L^−1^).

Kinetic Model	Parameters	Pb^2+^			Zn^2+^		
		Temperature, K					
		298	318	338	298	318	338
Pseudo-first order	*q*_exp_, mmol∙g^−1^	1.95	2.01	2.03	1.13	1.20	1.29
	*k*_1_, min^−1^	0.0036	0.0054	0.0087	0.0242	0.0334	0.0363
	*q*_e_, mmol∙g^−1^	0.384	0.378	0.332	0.697	0.697	0.617
	*R^2^*	0.856	0.997	0.990	0.991	0.964	0.958
	*E*_a_, kJ∙mol^−1^	18.69 ± 1.14			8.57 ± 1.27		
Pseudo-second order	*k*_2_, g·mol^−1^·min^−1^	322.9	270.7	580.0	257.4	275.9	457.5
	*q*_e_, mmol∙g^−1^	1.91	1.98	1.99	1.18	1.22	1.31
	*R^2^*	0.999	1.0	0.999	0.998	0.999	0.998
	*E*_a_, kJ∙mol^−1^	11.83 ± 0.72			11.82 ± 0.63		
Elovich	*α*, mmol·g^−1^·min^−1^	17757	17090	88258	4.13	5.16	5.96
	*β*, mmol·mg^−1^	8.70	8.45	8.90	6.46	6.03	6.07
	*R^2^*	0.944	0.957	0.859	0.928	0.940	0.945

**Table 7:** The selectivity of titanium phosphate for lead and zinc ions at a high concentration of Ca^2+^ ions (480 mg·L^−1^).

Metal	Initial Concentration, Mmol·L^−1^	*K*_d,_ 10^5^ mL·g^−1^
Pb^2+^	0.05	2.04 ± 0.53
	0.1	4.17 ± 0.42
	0.2	8.32 ± 0.37
Zn^2+^	0.1	1.28 ± 0.34
	0.2	2.45 ± 0.61
	0.3	3.89 ± 0.26
